# Assessing the feasibility of MOVING FORWARD, a multicentre transition intervention model across adolescent secure services in England: A cluster randomised feasibility trial

**DOI:** 10.1192/j.eurpsy.2022.602

**Published:** 2022-09-01

**Authors:** M. Livanou, R. Lane

**Affiliations:** Kingston University, Psychology, EE, United Kingdom

**Keywords:** adolescent secure hospitals, young people, transitions, cluster randomised trial

## Abstract

**Introduction:**

Young people moving from adolescent secure inpatient units to adult care in the UK present with multiple and complex needs and are more likely to experience poor transition outcomes related to enduring dependency on mental health services. However, there is lack of knowledge about the feasibility of transitional care models improving transition outcomes.

**Objectives:**

The MOVING FORWARD study aims to implement a new transition intervention model for young people transitioning from adolescent secure services to adult-oriented settings and test the feasibility of a future cluster trial measuring its effectiveness.

**Methods:**

The design of the study is a three-arm feasibility, cluster randomised controlled trial comparing the MOVING FORWARD intervention against standard transition preparation in six adolescent secure services.Young people between 17-19 years, their parents/carers and key workers will be allocated in two conditions and will receive four transition preparation workshops across six months. Data will be collected at three time points: (T0) baseline, (T1) 6-12 months post-intervention, and (T2)18-24 months post-baseline.

**Results:**

Thirteen young people and 17 staff members have contributed to the intervention design through online Advisory Groups. Common identified themes included appropriateness of module content and support during delayed transitions. An intra-class correlation coefficient will be calculated to inform the power of sample size. With a sample size of 50, we will be able to estimate a drop-out rate of 80%.

**Conclusions:**

This research will provide practitioners and policy makers with an evidence-based framework about barriers and facilitators to the proposed intervention and will enable services to reflect on quality of transitional care delivery.

**Disclosure:**

No significant relationships.

